# Tissue management in precision medicine: What the pathologist needs to know in the molecular era

**DOI:** 10.3389/fmolb.2022.983102

**Published:** 2022-10-26

**Authors:** Ricella Souza da Silva, Regina Pinto, Luis Cirnes, Fernando Schmitt

**Affiliations:** ^1^ IPATIMUP Diagnostics, IPATIMUP—Institute of Molecular Pathology and Immunology of Porto University, University of Porto, Porto, Portugal; ^2^ Department of Pathology, Faculty of Medicine of the University of Porto, University of Porto, Porto, Portugal; ^3^ CINTESIS@RISE (Health Research Network), Porto, Portugal

**Keywords:** molecular pathology, morphological control, oncology precision, personalized medicine, tissue management, solid tumors

## Abstract

Precision medicine is “an emerging approach for disease treatment and prevention that takes into account individual variability in genes, environment, and lifestyle for each person.” Among many medical specialists involved in precision medicine, the pathologists play an important and key role in the implementation and development of molecular tests that are in the center of decision of many therapeutic choices. Besides many laboratory procedures directly involved in the molecular tests, is fundamental to guarantee that tissues and cells collected for analysis be managed correctly before the DNA/RNA extraction. In this paper we explore the pivotal and interconnected points that can influence molecular studies, such as pre-analytical issues (fixation and decalcification); diagnosis and material selection, including the calculation of nuclei neoplastic fraction. The standardization of sample processing and morphological control ensures the accuracy of the diagnosis. Tissue or cytological samples constitutes the main foundation for the determination of biomarkers and development of druggable targets. Pathology and precision oncology still have a long way to go in terms of research and clinical practice: improving the accuracy and dissemination of molecular tests, learning in molecular tumor boards for advanced disease, and knowledge about early disease. Precision medicine needs pathology to be precise.

## 1 Introduction

Pathology is evolving to meet patient needs and be a central driver of personalized healthcare. The main objective of personalized medicine is to identify patients who are candidates for specific treatments. The individualized approach has transformed the oncological therapeutic scenario (Russo et al., 2022) and prognostic evolution (Siegel et al., 2021).

Traditionally, pathologists have been at the forefront of cancer diagnostics with knowledge of cancer biology, morphology and prognosis (Ooft et al., 2021). In precision medicine, target-based classification is progressively used to integrate the histology-based classification of tumors, which remains the pillar of cancer diagnosis and management (Danesi et al., 2021).

Precision oncology reveals an accelerated pace, with great financial investment and a rapidly growing pharmacological arsenal. The dynamic and changing environment has allowed gaps in the standardization and validation of molecular diagnostic procedures. Pathology plays an important role in the implementation and development of molecular tests—precision medicine needs pathology to be precise.

The involvement of the pathologist in genomic medicine and biomarkers starts with the management of the primary sample (Moore et al., 2018). Therefore, it is critical that the training pathologist understand the framework and basic technical elements of the molecular tests. Its performance, in accordance with current practices and guidelines for the molecular diagnostics workflow, also encompasses the main following phases: pre-analytical; diagnosis and appropriate selection of tissue sample (quality and quantity); use of “*in situ*” based techniques; integrate the molecular data in the original diagnostic report (translation to the clinicians); and educational activity (Schmitt, 2011; Cree et al., 2014; Rekhtman and Roy-Chowdhuri, 2016; Fassan, 2018; Roh, 2019; Gullo et al., 2021; Ooft et al., 2021; Vranic and Gatalica, 2021).

In this paper, we explore the pivotal and interconnected points that can influence the molecular study, such as pre-analytical issues (fixation and decalcification); diagnosis and material selection, including the calculation of nuclei neoplastic fraction. The standardization of sample processing and morphological control ensures the accuracy of the molecular diagnosis.

## 2 Pre-analytical

Suboptimal processing can alter morphological, immunohistochemical and molecular characteristics of histological and cytological samples (Bass et al., 2014; Gullo et al., 2021). The magnitude and direction of effects associated with a given pre-analytical factor are dependent on the object of analysis, whether DNA, RNA or protein (Penault-Llorca et al., 2022).

For all samples, the primary pre-analytical factors whose thresholds somehow affect molecular results can be grouped in: cold ischemia, fixation, decalcification and time of paraffin block storage (Bass et al., 2014; Cree et al., 2014).

Cold ischemia is the time that occurs between the removal and fixation of tissue and is especially problematic for large surgical specimens. It affects gene expression at RNA and protein levels and mutation analysis at DNA level, thus being a major factor in molecular pathology (Cree et al., 2014). The recommended thresholds for maximum cold ischemia time are: DNA <1 h for FISH, ≤24 h for PCR; RNA <12 h; Protein <12 h; Morphology <6 h (Bass et al., 2014).

The fixative worldwide accepted for tissue sample preservation is neutral buffered formalin (NBF). NBF penetrates tissue at around 1 mm/h, and fixation will only start when penetration occurs. NBF should only be used 24 h after dilution to 4% w/v, in order to reduce the effect of polymerization, and guarantee a stable 4% concentration. For cytological samples, fixation is commonly achieved with alcoholic-based fixatives (Cree et al., 2014). As the cold ischemia time, also the duration of fixation influences downstream nucleic acid, protein and morphological analyses (Penault-Llorca et al., 2022). The thresholds recommended for fixation times are: DNA <72 h; RNA 8–48 h; Protein 6–24 h; Morphology <1 year (Bass et al., 2014). An optimal fixation window of 6–48 h is recommended, based on findings that minimal nucleic acid degradation is observed before 72 h (Penault-Llorca et al., 2022). This period of time is also recommended to preserve the protein expression detected by immunohistochemistry. Sometimes, immunohistochemistry is used to detect anomalous expression or absence of expression as result of a molecular alteration (for example: P53 expression or absence of one of the mismatch repair proteins).

Decalcification is frequently not mentioned in the pathology report, though it can severely affect molecular analysis. To preserve the integrity of the nucleic acids and proteins, areas suspected of neoplasia in bone pieces should be processed without decalcification whenever they can be isolated. For smaller specimens, as bone biopsies, it is recommended to use Ethylenediaminetetraacetic (EDTA) as a decalcifying agent (Bass et al., 2014; Penault-Llorca et al., 2022).

Regarding the duration of paraffin block storage, the following thresholds were demonstrated: DNA ≤5 years; RNA ≤1 year; Protein ≤25 years for IHC, < 10 years for platforms requiring protein extraction (Bass et al., 2014).

## 3 Diagnosis and material selection

During the microscopic examination the pathologist should correlate the clinical information with morphology and recognize if there is the need for molecular analysis. The use of tissue in small specimens should be rational, avoiding unnecessary sections and complementary studies (histochemical immunohistochemical, or hybridization). In some countries, as in our experience, molecular studies are done in central laboratories that received material from different hospitals. In this situation is advisable to have a dedicated molecular pathology that evaluates the available material before proceeding to any molecular study.

The following criteria are relevant in this phase: 1) Slide/sample for molecular analysis must accurately represent the diagnosis of the pathological report; 2) Most representative slide/sample: show all the characteristics of the neoplasm such as morphological type and staging; 3) Avoid samples with extensive areas of tumor necrosis, inflammatory infiltrate and fibrosis; 4) It is recommended that the pathologist marks the area of the section containing neoplasia on the hematoxylin and eosin (H&E) slide at the time of diagnosis.

To avoid gross sampling errors, the pathologist must directly certify that the block sent for analysis corresponds to the patient and the diagnosis. In patients with multiple samples, the most recent tissue should be used (Russo et al., 2022). It is also important to avoid choosing samples with scarce number of tumor cells or material previously used for a large number of stainings and/or molecular tests. The goal is to ensure that the sample that will be submitted to molecular tests be the most representative qualitatively and quantitatively, since the morphological control depends on these factors.

In our molecular laboratory, a specific and validated protocol of cutting sections has been optimized and is currently in use. First we perform sections that will be used for extraction of nucleic acids—at 10 μm thickness, and only after we do a 3 μm section and stain with H&E for morphological evaluation. The quantity of extraction sections obtained varies according to the molecular assay and/or biomarker: nine sections for Next Generation Sequencing (NGS) panels requiring DNA and RNA isolation (e.g., EGFR, ALK, ROS1, NTRK, BRAF, RET, MET, ERBB2/HER2, NRG1, and FGFR1); 4–6 sections for Sanger sequencing studies requiring only DNA isolation (e.g., BRAF, PIK3CA, MSI or methylation studies) four sections for real-time PCR based studies (e.g., RAS); 3–4 sections for studies requiring only RNA isolation (e.g., NTRK, PAM50).

## 4 Diagnosis and select of correct material: Morphological control for molecular testing

The percentage of neoplastic cells present in the selected sample must be estimated for morphological control (Cree et al., 2014; Dufraing et al., 2019; Gullo et al., 2021). Tissue quality is expressed as the percentage of neoplastic cells to the total number of nucleated cells in a sample.

Recommendations for morphological control: 1) The evaluation of the percentage of malignant cells corresponds to the percentage of malignant nuclei (Gullo et al., 2021); 2) The evaluation of the percentage of malignant cells does not correspond to the size/area of the neoplasm; 3) It is recommended to make the estimation in deciles (e.g., 10%, 20%, …, 50%, … 100%) (Pei et al., 2019).

In estimating the percentage of viable malignant nuclei, regions with necrosis or inflammatory cells or desmoplastic stroma or mucus should be avoided and excluded for molecular analysis. If this is not possible, it is necessary to consider that these non-neoplastic nuclei are viable and dilute the percentage amount of malignant neoplastic DNA (Cree et al., 2014; Dufraing et al., 2019; Gullo et al., 2021; Kotoula et al., 2021). Tumor heterogeneity also needs to be taken into account when evaluating the percentage of neoplastic cells. These different areas, which represent cancer-specific growth patterns and tumor grading variation, must be present in the sample that will be submitted for molecular testing (Cree et al., 2014; Jennings et al., 2017; Nicholson et al., 2022).

The macrodissection is an important tumor cell enrichment technique, highlighting the critical role of the pathologist. This technique improving the accuracy of the molecular analysis, for direct sequencing or next generation sequencing (Gullo et al., 2021). It is considered necessary in the majority of sections from large tumor surgical specimens and occasionally in biopsy sections. The procedure is performed on unstained deparaffinized sections containing tissue fragments that have been directly processed for DNA/RNA extraction (Kotoula et al., 2021). The laser capture microdissection is largely a research tool and not necessary for routine molecular pathology (Cree et al., 2014).

Small samples with limited tumor cell content (<30%) may permit morphological classification; however, the quantity of tumor tissue is not always sufficient for biomarker testing (Penault-Llorca et al., 2022). The minimal amount of tumor DNA/RNA, as well as the minimal malignant cells required for molecular testing, are variable and dependent on the analytic sensitivity of a particular molecular assay (Dufraing et al., 2019). In general, a fraction of malignant cells greater than 10%–20% is considered a lower acceptable limit for molecular methods (e.g., 20% for Sanger sequencing or ∼10% for next-generation sequencing).

If samples have lower than acceptable levels of malignant nuclei or in the absence of viable tumour tissue, histological slides used for morphological diagnosis (H&E and immunostained slides) can also be an additional alternative, to extract material for molecular analysis. From our experience, we can extract DNA and RNA from previous H&E slides, while the use of previous immunostained slides is indicated only for DNA extraction. RNA obtained from previous immunostained slides, shows degradation and does not give viable results. The utilization of the archive slides must be reserved and discussed case by case. Furthermore, the scanning and digital archiving of the slides is mandatory to ensure medical-legal issues.

When DNA is purified from a pure tumor cell population (100% tumor cellularity), the mutant allele frequency would be expected to be 50%. If a sample harbored 50% tumor cells and 50% non-tumor cells, the latter population harboring two copies of the wild type allele, the mutant allele frequency would be 25% (Roh, 2019). If in the purified nucleic acid sample, the mutant allele represent 15%–20% of the allelic population overall (mutant plus wild type), it means that the sample has approximately 30%–40% tumor cells.

It is important to note that an intact diploid cell produces 6–7 pg of DNA and 10–30 pg of RNA. Input nucleic acid mass requirements for molecular testing are variable, with minimum recommendations ranging from of 1–10 ng (typical minimum input for most NGS platforms), 50 ng, 100 ng, and 200 ng (Benayed et al., 2019; Roh, 2019; Qu et al., 2020; Penault-Llorca et al., 2022).

Cytopathology samples ethanol-fixed, as smears and touch preparations, usually contain higher-quality nucleic acids than formalin-fixed, paraffin-embedded (FFPE) samples and are useful for molecular testing (Gan and Roy-Chowdhuri, 2020). For example, to isolate 10 ng of nucleic acids approximately three– fourfold more cells are required from a FFPE sample than from ethanol-fixed material. It is beneficial if the pathologist indicates the areas containing the highest proportion of neoplastic cells on the slide because it helps to avoid contamination of the material for nucleic acid extraction by non-neoplastic elements (Dietel et al., 2016). Particularly in cytology material, preliminary experiences have demonstrated that samples with <100 cells are not suitable for NGS, samples between 100–2000 represent low levels and >5000 cells are suitable for any NGS including large panels.

**FIGURE 1:**
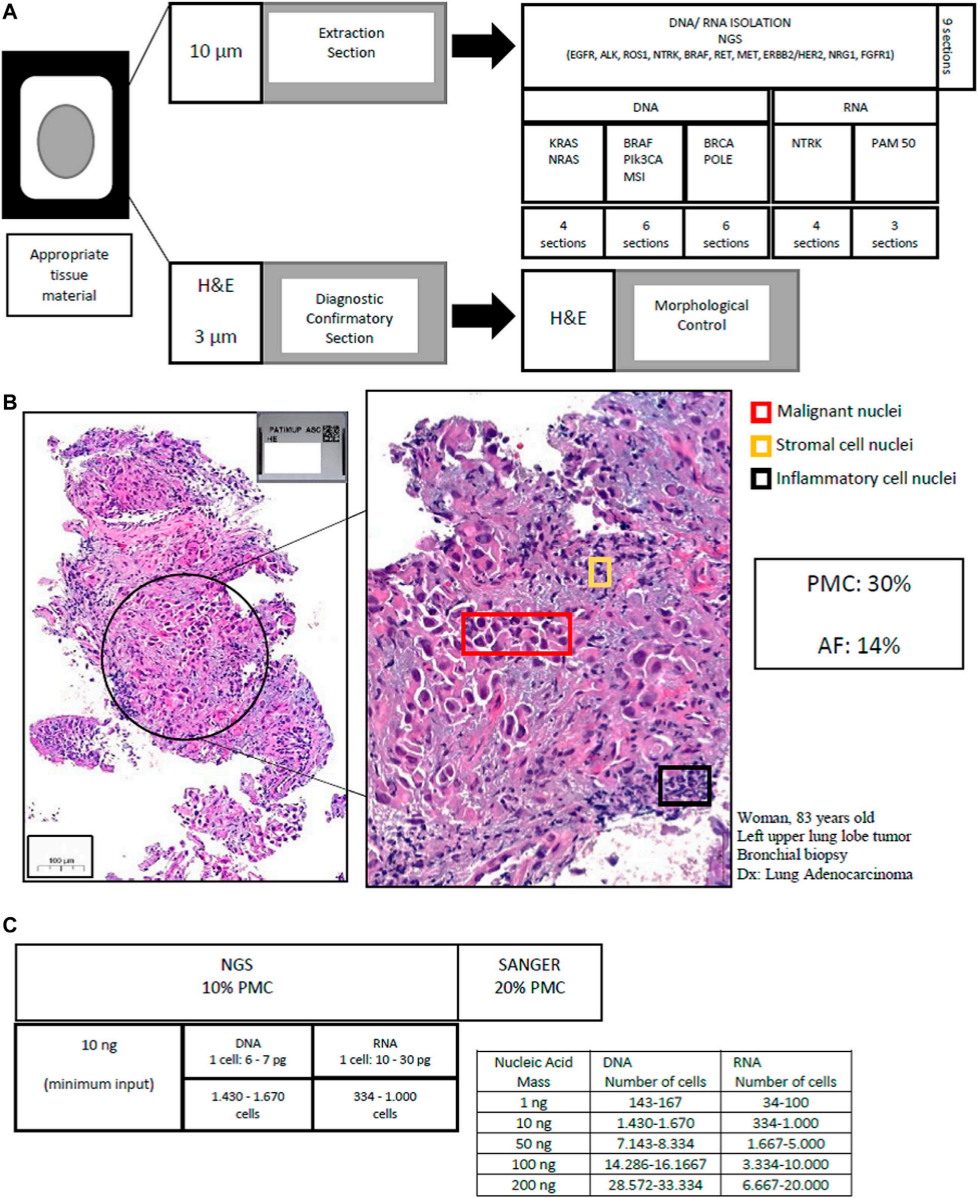
Workflow to sample processing and morphological control in molecular pathology. **(A)** Tissue cutting protocol (FFPE or cytological preparations): DNA/RNA extraction and H&E stain for morphological control. **(B)** Morphological Control in lung adenocarcinoma: The percentage of neoplastic cells corresponds to the percentage of malignant nuclei. Pathological assessment that 30% of the malignant cells were viable. The allele frequency verified was 14%, confirming the estimate. **(C)** Number of cells needed by nucleic acid quantification in single-multi-gene assays. PMC (percentage malignant cell), AF (allele frequency), NGS (next generation sequencing).

Figure 1 shows the approach from sample processing to morphological control in molecular assays.

The interobserver variability in estimating the percentage of neoplastic cells is 20% (Dufraing et al., 2019) and pathologists more accurately estimate the percentage of malignant nuclei in cases containing low amounts of tumor cells (Viray et al., 2013). The variability in assessment can be minimized through feedback from the sequence variants with a review of the histomorphology and assessment of biomarkers. The tumor percentage estimation and allele frequency (AF) should be compared (Ooft et al., 2021). When AF is higher than expected, the variant might be a germline, or the gene might be affected by heterozygosity or tumor cell aneuploidy might have occurred. If a low AF is detected in a sample containing a high percentage of neoplastic cell, the result could be interpreted as a testing artefact or as an indicator for subclonality of a given gene in the context of tumor heterogeneity (Dufraing et al., 2019).

**FIGURE 2:**
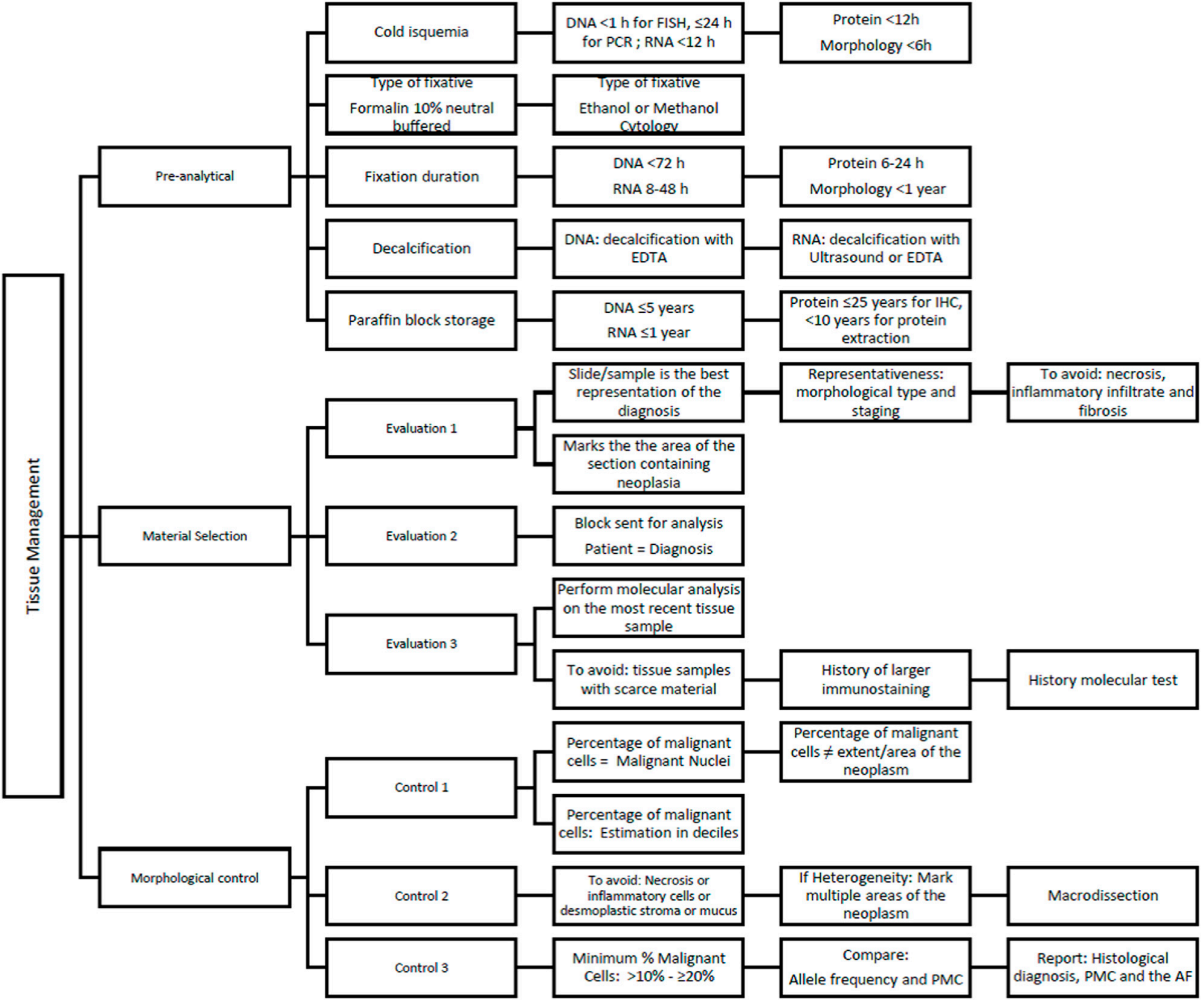
Recommendations for Solid Tumors Tissue Management.

The pathology molecular report should include the histopathological diagnosis, the estimated percentage of malignant cells present in the tissue used for DNA/RNA extraction, and the allele frequency. Figure 2 describes the recommendations for solid tumors tissue management for molecular analysis.

## 5 Discussion

Diagnosis, classification, prognosis (Ooft et al., 2021) and even study of mechanisms of cancer development (Echejoh et al., 2021) are the core of pathology. The development of precise medicine brings molecular diagnosis for the prime time. Pathologists are probably the most appropriate for connecting morphology, clinical setting, mutational status, and the reflection of these findings in therapeutics. Previous tumor categorization criteria are being replaced by a new approach based on specific genetic abnormalities, in which tissue or cytological samples constitutes the main foundation for the determination of biomarkers and development of druggable targets.

The standardization of tissue sample handling at each step—sample processing and morphological control—ensures the accuracy of the diagnosis. Estimation of percent neoplastic cellularity can also affect care in other diagnostic settings, including assessment of copy number variation, chromosomal translocation, determination of residual cancer burden after neoadjuvant therapy, and other genomic aberrations that can be affected by contaminating normal DNA (Viray et al., 2013).

Best practice guidelines aim to management tissue and support a complete molecular diagnosis, so that eligible patients may benefit from targeted therapy. A biomarker negative sample with a tumor cell content below the thresholds for analysis should be determined as inconclusive, requiring further assessment. Consequently, the proportion of cells may also inform the choice of molecular testing.

Molecular therapy demonstrates remarkable response rates, making advances in reducing cancer mortality (Siegel et al., 2021), although the response to genome-targeted therapy has been modest (2.73% in 2006 to 7.04% in 2020) and more trials are needed to determine the impact on survival, which currently stands at 4.7 months (Haslam et al., 2021; Haslam et al., 2022).

In this scenario, it is necessary to continue researching the complex interaction of tumor biology, microenvironment, and immune response; to disseminate molecular tests in clinical practice; to encourage molecular tumor board for advanced diseases; and to learn about early diseases. Pathology and oncology in precision medicine still have a long way to go in research and clinical practice.
